# Supplementary figures and images for: Patterns of selection against centrosome amplification in human cell lines
Source: PLoS Comput Biol. 2021 May 12;17(5):e1008765. doi: 10.1371/journal.pcbi.1008765 (PMC8143425; doi:10.1371/journal.pcbi.1008765)

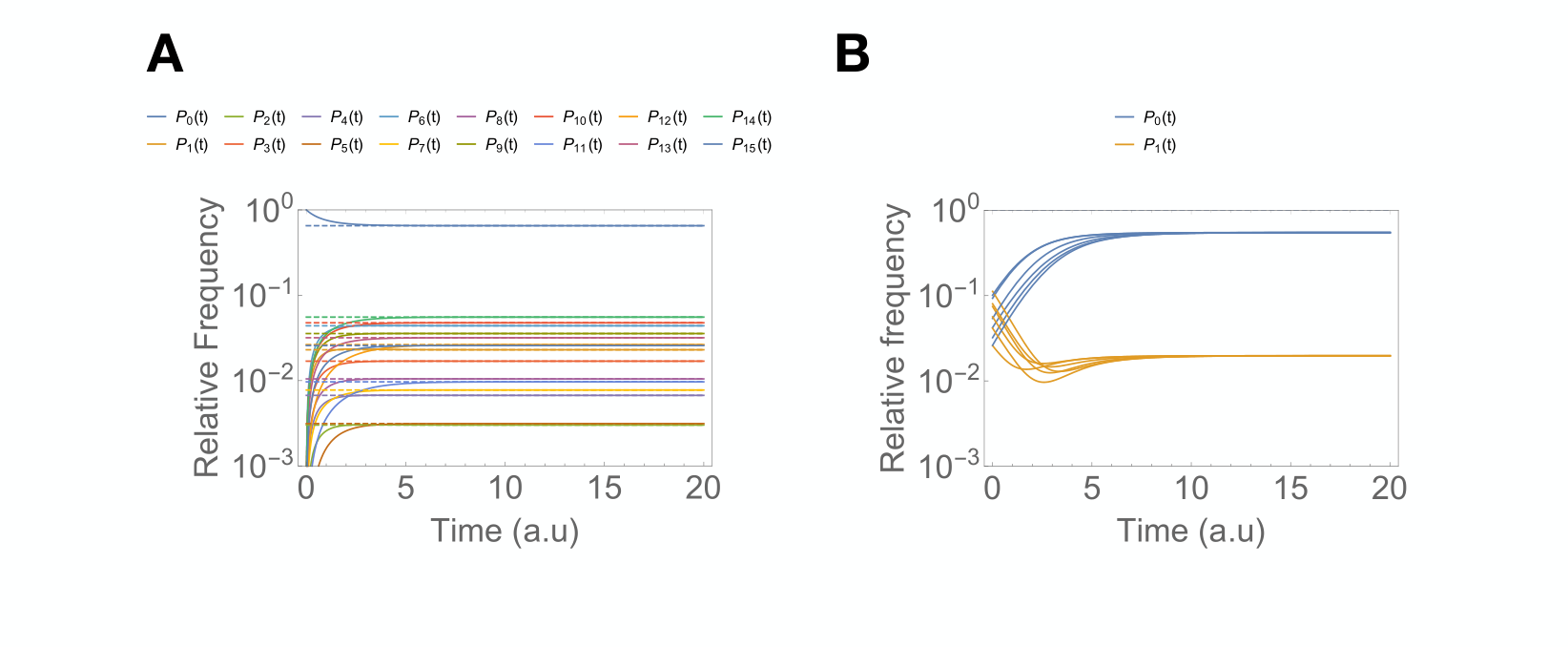

Supplement: S1 Fig — A—Comparison between numeric integration of the general model and the corresponding equilibrium expression (2), evaluated at the same parameter values. We assumed imax = 15 and generated pseudo-random parameter values for all ri and μi,j. B—Comparison between numeric integration of the general model from different initial conditions and the corresponding equilibrium expression. We generated a set of pseudo-random parameter values for all ri and μi,j and initial conditions. Note that the y-axis is in log-scale. (TIF) [file pcbi.1008765.s001.tif]

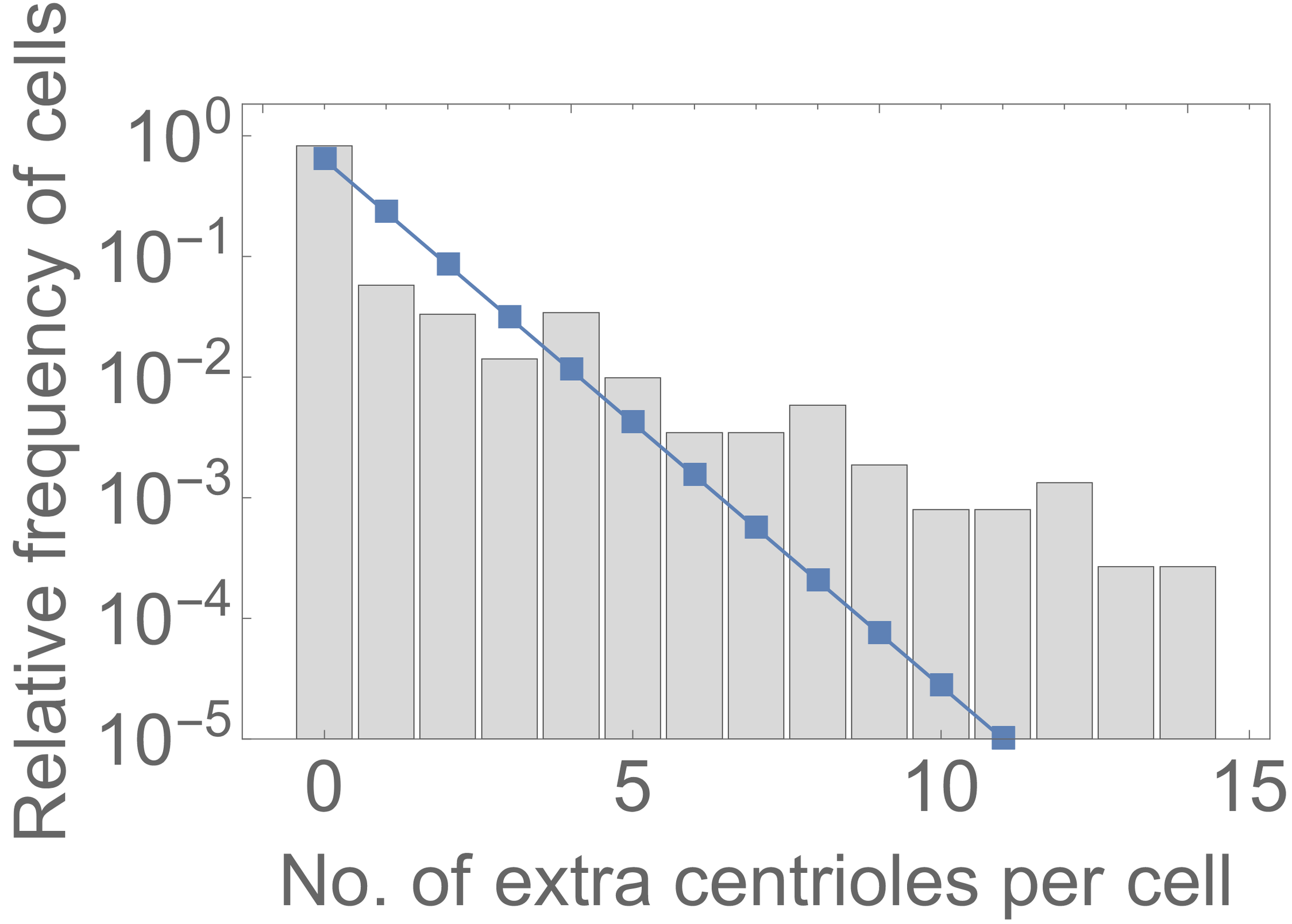

Supplement: S2 Fig — Best fitting geometric distribution (blue) to the pooled distribution of centriole numbers in all sampled populations. Number of sampled cells: n = 3746. (TIF) [file pcbi.1008765.s002.tif]

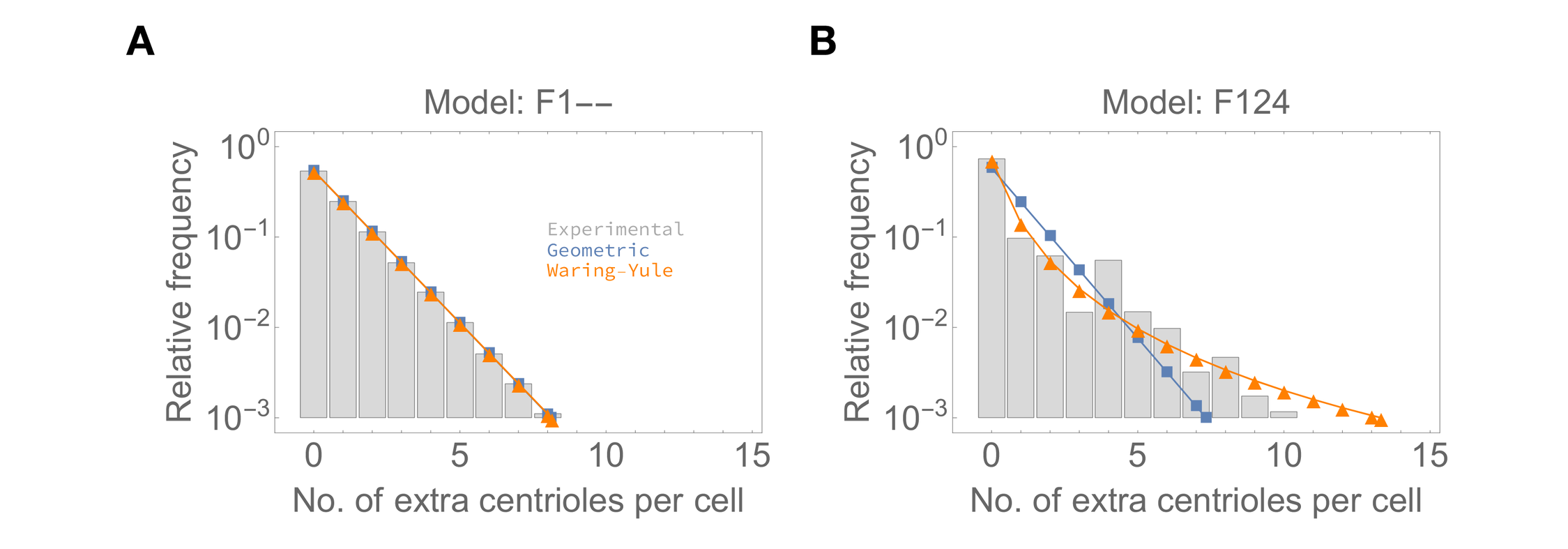

Supplement: S3 Fig — Best fitting geometric (blue) and Waring-Yule (orange) distributions to 1,000,000 simulated data points (relative frequencies indicated as grey bars). A—Data simulated from model F1-- (r = −0.3, μ1 = 0.6). B—Data simulated from model F124 (r = −0.5, μ1 = 0.2, μ2 = 0.1, μ4 = 0.1). Data points were generated by multinomial sampling from the equilibrium distributions evaluated at the indicated parameter values. Note that the y-axis is in log-scale. (TIF) [file pcbi.1008765.s003.tif]

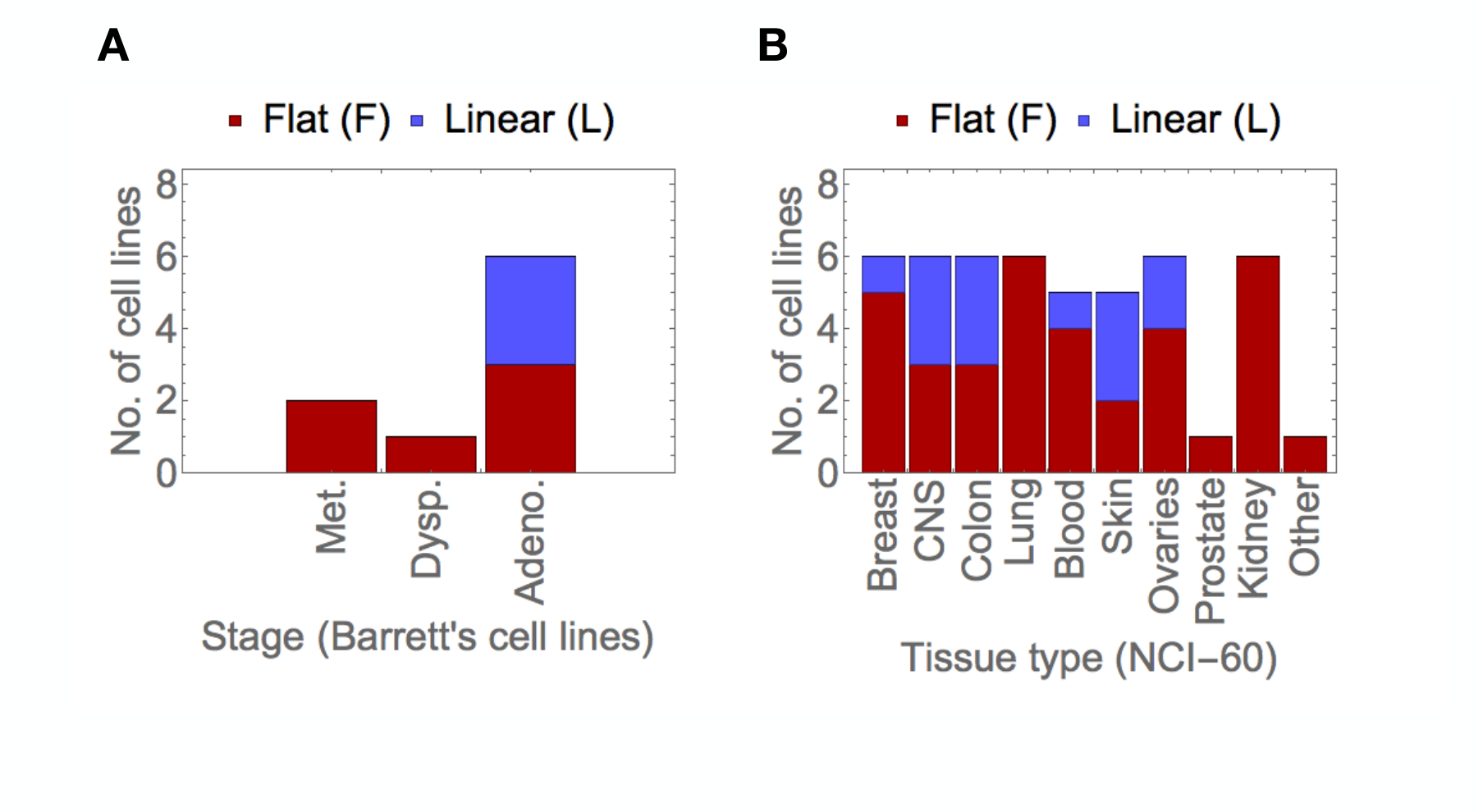

Supplement: S4 Fig — Models sharing the fitness function of the best model for each cell line in the data sets. A—Barrett’s esophagus data set, grouped by developmental stage. B—NCI-60 data set, grouped by tissue type (including cancer and non-cancer cell lines). “Other” refers to an RPE cell line that was used as a control. (TIF) [file pcbi.1008765.s004.tif]

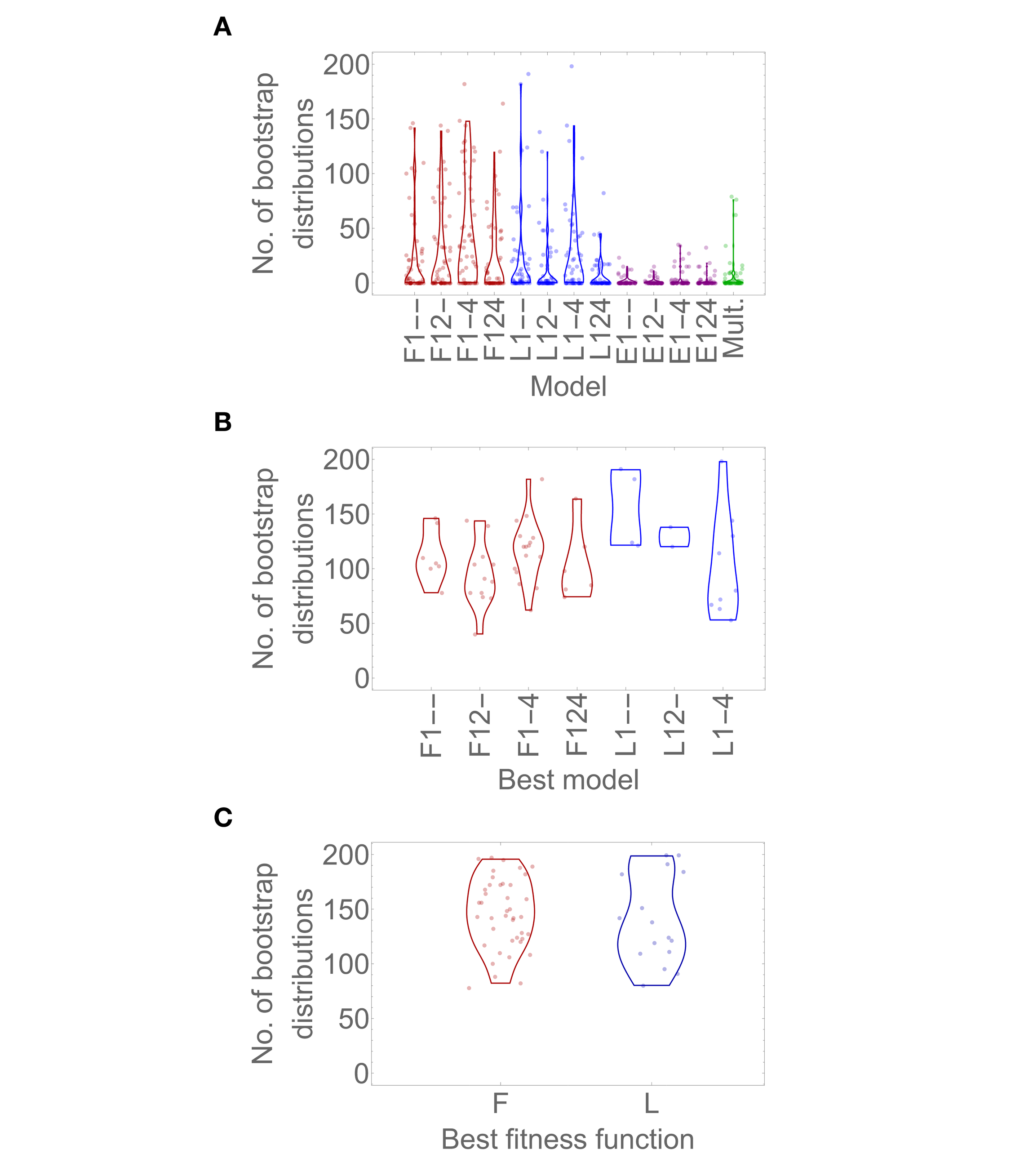

Supplement: S5 Fig — A—Number of bootstrap distributions (out of the 200 generated for each cell line) explained by each model. We obtained indistinguishable BIC scores for multiple models in 381 bootstrap distributions spread across 26 different cell lines (“Mult.”). C—Number of bootstrap distributions explained by models sharing the same fitness function as the best model for each cell line. The fitness functions of the models are indicated in red (flat), blue (linear), purple (power-law), and green (multiple models). (TIF) [file pcbi.1008765.s005.tif]

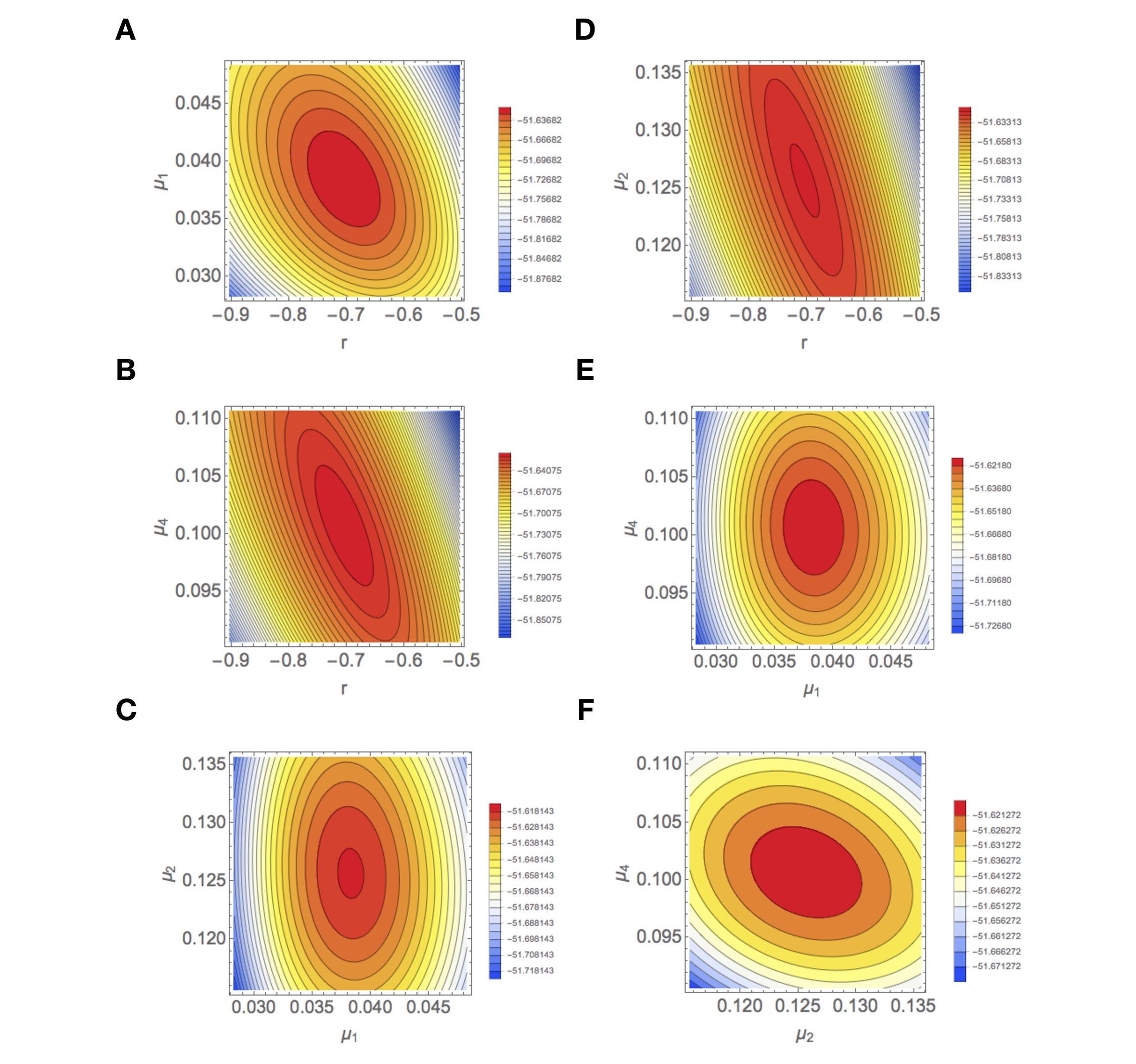

Supplement: S6 Fig — We fitted the model to a random empirical distribution from the analysed data sets and calculated the likelihood values centered around the maximum as a function of pairwise combinations of parameter values. A—r and μ1; B—r and μ2; C—r and μ4; D—μ1 and μ2; E—μ1 and μ4; F—μ2 and μ4. Note that the scale for r is different from that of the centriole overproduction parameters μ1,μ2, and μ4. (TIF) [file pcbi.1008765.s006.tif]

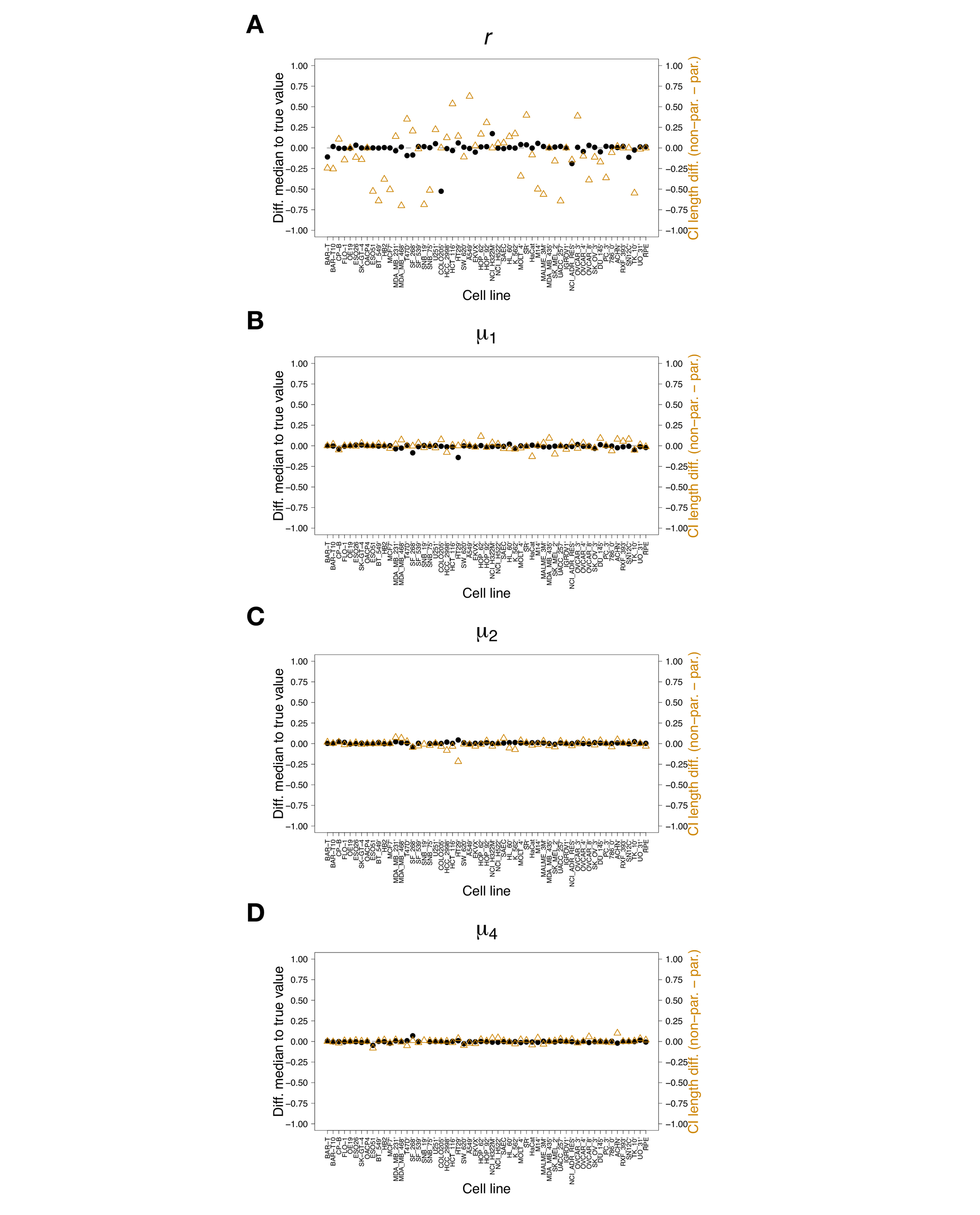

Supplement: S7 Fig — Difference between the median value of the parametric bootstrap distribution for each parameter value and the input value for the simulated data (in black) and difference between the confidence interval length of the non-parametric and parametric bootstrap distributions (in yellow). A—r. B—μ1. C—μ2. D—μ4. (TIF) [file pcbi.1008765.s007.tif]
