# Supplementary material for: Patterns of selection against centrosome amplification in human cell lines
Source: PLoS Comput Biol. 2021 May 12;17(5):e1008765. doi: 10.1371/journal.pcbi.1008765 (PMC8143425; doi:10.1371/journal.pcbi.1008765)
Supplement: Attachment — Submitted filename: response_letter_final_v.pdf [file pcbi.1008765.s008.pdf]

Dear Jacopo Grilli and Douglas Lauffenburger,

Thank you for mediating the peer review process of our manuscript. We would like to thank the reviewers for their excellent feedback that has helped us improve our manuscript.

In the following we respond in detail to each comment (with our comments highlighted in green), including a transcript of any changes to the text (also highlighted in green in the manuscript).

With the best regards,
Marco António Dias Louro, Mónica Bettencourt-Dias, and Claudia Bank

Comments to the Authors:
Please note here if the review is uploaded as an attachment.
Reviewer #1: In this manuscript Marco et al., have collected centriole amplification data from two previous publications that quantitated the number of centrioles in various cell lines. Based on the number of centriole distribution authors have tried to fit models that could best explain the centriole distribution in the individual cell line population. Based on their model (and its obvious too) authors state that there is selection against centriole amplification irrespective of their origin. The methods are in detail and manuscript is very well written. Below are my suggestions to improve this manuscript further.

We thank the reviewer for their positive assessment of our manuscript.

The abstract starts with “The presence of extra centrioles, or centrosome amplification, is a hallmark of cancer.”. We know that cancerous cells have uncontrolled division/growth. The manuscript would read better by including discussion on “how cancer cell evades the bias against centriole amplification”?

Ask editor if we can ask reviewer what this means

Reply: We agree that this is an important aspect to be discussed. To our understanding, we have addressed how cancer cells cope with extra centrioles both in the Introduction and Discussion sections:

(l. 46)
“However, some mechanisms are known to provide protection against centrosome amplification. For example, centrosome clustering mechanisms allow cells to group extra centrioles in two spindle poles, thus improving the viability of daughter cells (13, 14, 16).”

(l. 385)
“To this day, the contribution of centrosomal anomalies to cancer development remain controversial, with some studies showing that higher numbers, via Plk4 overexpression, can initiate or aggravate tumorigenesis (35, 36), and others showing that it is not sufficient and may even slow down progression (37, 38). On the other hand, extra centrioles are

associated with other cancer hallmarks, such as aneuploidy (14, 39) and invasion (40-42), and often correlate with a more aggressive cancer phenotype (3, 7).”

(l. 396)
“For example, in the case of the Barrett's esophagus progression model, the increase in centriole numbers from the metaplasia to the dysplasia stages can be explained by loss of p53 (9). This can be interpreted as a reduction in the strength of negative selection, since p53 can lead to cell-cycle arrest or cell death in the presence of extra centrioles. If this is true, it would be interesting to quantify how strong the decrease in selective pressure and if it is sufficient, by itself, to account for the shift in centriole number distributions.”

In NCI 60 cell line panel there are many cell lines originating from solid tumors as well as non-solid tumors (though a handful). Was there any difference in centriole amplification distribution between cell lines derived from solid and non-solid tumors? Though I do see one example in Figure 1.

Reply: We appreciate this comment as we had not considered differences between solid and non-solid tumours. We assume the reviewer refers to non-leukemia (solid) and leukemia cell lines (liquid). If that is the case, we take it to be a particular case of the more general question regarding tissue of origin (addressed below). In short, there is no model selection pattern distinguishing solid vs. non-solid tumours. Furthermore, the estimation errors do not allow us to distinguish between different cell lines.

In Figure 1D one fourth of the total cell lines do not show any difference between geometric and Waring-Yule distribution. On the other hand, one fourth of cell lines show relatively very high (ranges 10 to ~40) delta chi square values. What authors have to say about this? What are those cell lines at the two extreme ends? Please discuss about the features of these two groups of cell lines (if any) in the relevant section. Also, where are the five non cancer cell lines on this graph. Do the non-cancerous cell lines show different pattern? If the non-cancerous cell lines show similar pattern like cancerous cell lines then this should be mentioned or shown as supplementary figure.

Reply: We thank the reviewer for their questions. Cell lines with higher delta-chi-squared values simply identify empirical distributions where cells with high centriole numbers are overrepresented with respect to a geometric distribution, and the opposite for cells with low delta-chi-squared values. In addition, the group of cells with low delta-chi-squared values also includes cell lines whose sampled population includes very few cells with centrosome amplification, in which case the geometric and Waring-Yule distributions are indistinguishable. We labeled the five control cell lines used in the NCI-60 study and modified the corresponding paragraph such that it reads:

(l. 136)
“Visual inspection of model fits suggests the Waring-Yule (heavy-tailed) distribution is a better fit to the represented empirical distributions (Figure 1A-C). In addition, our results indicate positive values of Delta X^2 for the majority of cell lines, suggesting a better fit of the Waring-Yule (heavy-tailed) distribution (Figure 1D). For 16 out of 67 cell lines, we obtained

values of Delta X^2 ~= 0 (more accurately, <= 1), indicating exponential-like and not heavy tails. Although the control cell lines used in the NCI-60 study rank in the bottom half of cell lines ordered by ascending Delta X^2 value, apart from HaCat, they are not clearly separated from the remaining cell lines. Thus, our results suggest that a simple model reminiscent of classical mutation-selection balance, yielding a geometric distribution of centriole numbers in the population, fails to explain the data for most cell lines. However, it should be noted that some of the 16 cell lines included in the group with near-zero Delta X^2 values contain few cells with centrosome amplification in the sampled population (e.g. OACP4 - 1 out of 61 cells with centrosome amplification; IGROV1 - 1 out of 58 cells with centrosome amplification), in which case there is little information to distinguish between geometric and Waring-Yule distributions."

Finally, the empirical distributions of centriole numbers per cell can be found in sections 2.3 and 4.3 of the adjoining code, and delta-chi-squared values for each cell line can be found in section 3.2.

In Figure S2 any underrepresentation of wild-type-like cell is not evident. The statement should be modified.
Reply: We thank the reviewer for taking notice, as the underrepresentation of cells with wild-type-like centriole numbers is indeed not visible in log-scale. Now, it simply reads:

(l. 126)
In contrast, we observed an overrepresentation of cells with high centriole numbers (Figure S2)

There is no discussion or any effort on splitting the data based on their tissue of origin, at least for those tissue types that have more than 5 cell lines to see if there is any significant difference between the tissue types.

Also compare different tumour types within the same tissue
Reply: The reviewer is raising an interesting question.. With respect to model selection, we refer the reviewer to Fig. S4, where we analysed model selection results by tissue type/cancer progression stage. Indeed, lung and kidney cell lines of the NCI-60 panel are universally best explained by models assuming a flat fitness function, whereas all other cell lines display a mix of flat and linear fitness functions. Regarding parameter estimates, the wide error margins do not allow to distinguish between cell lines, or between tissue types. Concordantly, we modified the following sentences in the main text:

(l. 210)
"Strikingly, the best models for 41 out of 57 cell lines assumed the flat fitness function, including all six lung and kidney cell lines in the NCI-60 panel, and the two metaplasia and one dysplasia cell lines in the Barrett's esophagus data set (see Fig. S4 for model selection results grouped by tissue of origin)."

(l. 238)

“However, the confidence intervals for both parameters are considerably wide, spanning almost the whole parameter range in the case of the intrinsic growth rate r, such that we cannot identify significant differences between cell lines or by tissue of origin.”

For Figure S3 please include more detail in the methods section, how exactly simulation was done.
Reply: We added the simulation details to the figure legend. It now reads:

(Fig. S3)
“Data points were generated by multinomial sampling from the equilibrium distributions evaluated at the indicated parameter values”.

In the discussion section authors state that “Interestingly, our analysis suggests that selection acts strongly against any number of excess centrioles in most cell lines. This means that deleterious effects arise as soon as excess centrioles are produced, whereas the actual number does not seem to matter for selection.”. If the actual number of centrioles does not matter then they should see the same frequency across all additional number (compared to WT) of centrioles. However, based on their result this is not the case. Please explain.
Reply: We thank the reviewer for raising this issue. Indeed, that would be the case if all numbers would be equally accessible. Consider the simplest model, F1--. Extra centrioles are produced in a stepwise manner, such that the sub-population of cells with one extra centrioles is produced from the sub-population with wild-type numbers, the sub-population with two extra centrioles is produced from the one with a single extra centriole, and so on and so forth. Thus, if the wild-type-like population is the most abundant one (as is the case for all of the analysed cell lines), the second most-abundant one will be the sub-population with one extra centriole, then the sub-population with two, etc. We have clarified this matter in the Discussion section:

(l. 327)
“This means that deleterious effects arise as soon as excess centrioles are produced, whereas the actual number does not seem to matter for selection, and that the shape of the distribution is determined chiefly by the mechanism(s) of centriole overproduction.”

Reviewer #2: Review of Patterns of selection against centrosome amplification in human cell lines, by Dias Louro et al., submitted to PLOS Computational Biology

This interesting paper uses model generation and fitting to data to determine how extra centrioles are produced and selected against in cancer and pre-cancerous cells. The topic is biomedically important, and the authors’ approach, on the whole, very well reasoned and carefully laid out. The choice of a deterministic formalism is commendable. The variables in it, if need be, can be interpreted as frequentist probabilities. It is interesting to see novel, distinct mutation-selection distributions arising in this instance of extra-genetic (in the narrow sense of DNA) inheritance. The model can serve as an example for mutation-selection of cell structural features that are inherited, with modification, through cell division. Although the present model is consciously constructed in such a simplified way as not to dwell on the

fitness and mutability consequences of the still incompletely understood cytoskeletal mechanisms that involve supernumerary centrioles, centriole number dynamics should still be a grateful subject for more mechanistically detailed modeling in the future, compared with other potential applications of this approach to cell structure inheritance. The conclusions reached here through sophisticated comparative model fitting – that there is a flat and low fitness landscape outside the basal centriole number – are not hard to accept, given what we know about abnormal mitoses. Notwithstanding, they are far from trivial and add to this still developing field. I have two minor remarks, and one major one.

We thank the reviewer for their positive assessment of our manuscript.

1 (major). Does the model presuppose that unequal division of centrioles between daughter cells never happens? Unequal division, which does occur (the paper refers to the relevant literature in the introduction), results in both an increase and a decrease of the centriole numbers in the progeny. Both are, in general, heritable. I believe that unequal division of the centriole number is essentially the norm past one extra centriole duplication. The Discussion section on limitations of this study speaks of "centriole segregation" as having been neglected; it appears the authors may be referring to the issue I am raising here. However, the meaning of segregation is somewhat obscure in that passage. If it is indeed argued that strong negative selection makes unequal distribution between daughter cells inconsequential, it would make equally inconsequential all mutations in the centriole number, other than those from the basal number. It seems that the model behavior that is evaluated against empirical data will be rather strongly affected by unequal centriole distribution between daughter cells.
Reply: The reviewer is raising an important issue, which is indeed what we referred to as "centriole segregation". We agree that unequal division of centrioles could affect model predictions significantly and we further elaborated on the reasons which lead us to opt against it. We rewrote the corresponding section in the Discussion in the following way:

(l. 367)
"Importantly, one of the aspects we simplified is how extra centrioles are distributed by the daughter cells after mitosis, or centriole segregation. Whereas after correct centriole duplication, each daughter cell inherits a single centriolar pair, it is still unclear how extra centrioles are distributed. However, while this manuscript was in review, Sala et al. reported that the degree of asymmetry in centriole segregation increases with the number of extra centrioles in RPE-1 cells transiently overexpressing Plk4 (31). Since most cells with centrosome amplification in the samples we have analysed contain only a few extra centrioles, it can be expected that centriole segregation is predominantly symmetrical. Moreover, this study further reports that extra centrioles are capable of duplication. Thus, cells should have the same number of centrioles as their mothers on average. In light of these results, we conclude that our deterministic approximation is reasonable."

Regardless, the key feature in our modelling framework is that it can generate stable equilibrium distributions. Since our data set includes cells with abnormally high centriole numbers, we assumed the existence of centriole overproduction that is counteracted by negative selection. Note that whereas both overproduction and selection affect the total

number of centrioles in the population, centriole segregation does not - the sum of the number of centrioles in the daughter cells should equal that of the mother. Thus, explicit centriole segregation is neither necessary nor sufficient to generate a stable equilibrium. In conclusion, we acknowledge that centriole segregation should play an important role in shaping the distribution of centriole numbers in proliferating cell populations. The detailed dynamics of centriole segregation should be addressed in the future with the acquisition of time-series data and individual-based modelling.

2 (minor). The steady-state solution is said to be locally stable. The methods suggest that this is an observation based on convergence from randomly sampled states. How do we know it is not globally stable? In the absence of analysis, stating simply that the model converges to this solution from randomized initial conditions may be preferable, although this is normally taken as evidence of uniqueness and global stability of the solution. It seems to me that the model, based on its form, should have only one time-independent solution, absent exotic parameter and initial condition choices.

Reply: We agree with the comment and modified the text to better reflect our approach:

(l. 101)
"We propose the following expression that describes a fully polymorphic equilibrium distribution, i.e. allowing cells of any sub-population to occur (see Figure S1 for a numerical example and a test of convergence from random initial conditions; see also Methods), for an arbitrary value of i_{max}*"*

(l. 416)
"Then, we verified if the expression was correct by comparing it to the steady-state obtained from numerical integration of equation Eq. (1), and further confirmed that the system converges to the same equilibrium for random initial conditions (Figure S1)."

3 (minor). Mutation-selection literature references might help the reader where this approach is first mentioned. Similarly, the reader will be helped by an explicit literature reference to the original quasi-species model (for primordial biopolymer) that had the discussed type of flat fitness landscape.

Reply: We added the suggested references.

(l. 98)
"This model is the continuous-time equivalent of the model proposed by Moran (27) in the absence of back mutation, and included in the framework of the original quasispecies model quasispecies model (28-30)."

(l. 336)
"Second, simple models incorporating single-centriole overproduction events and a constant fitness function (i.e. akin to the classical formulation of mutation-selection balance in population genetics and widely explored in quasispecies models (reviewed in (33)) were sufficient to explain the shape of the centriole distribution in a few cell lines, whereas in

others a more complex relationship between selection and overproduction improved the fitting."
